# Supplementary material for: A Shared Pathogenic Mechanism for Valproic Acid and SHROOM3 Knockout in a Brain Organoid Model of Neural Tube Defects
Source: Cells. 2023 Jun 23;12(13):1697. doi: 10.3390/cells12131697 (PMC10340169; doi:10.3390/cells12131697)
Supplement: Supplementary file 1 [file cells-12-01697-s001.zip › Supplemental Figures V3.pdf]

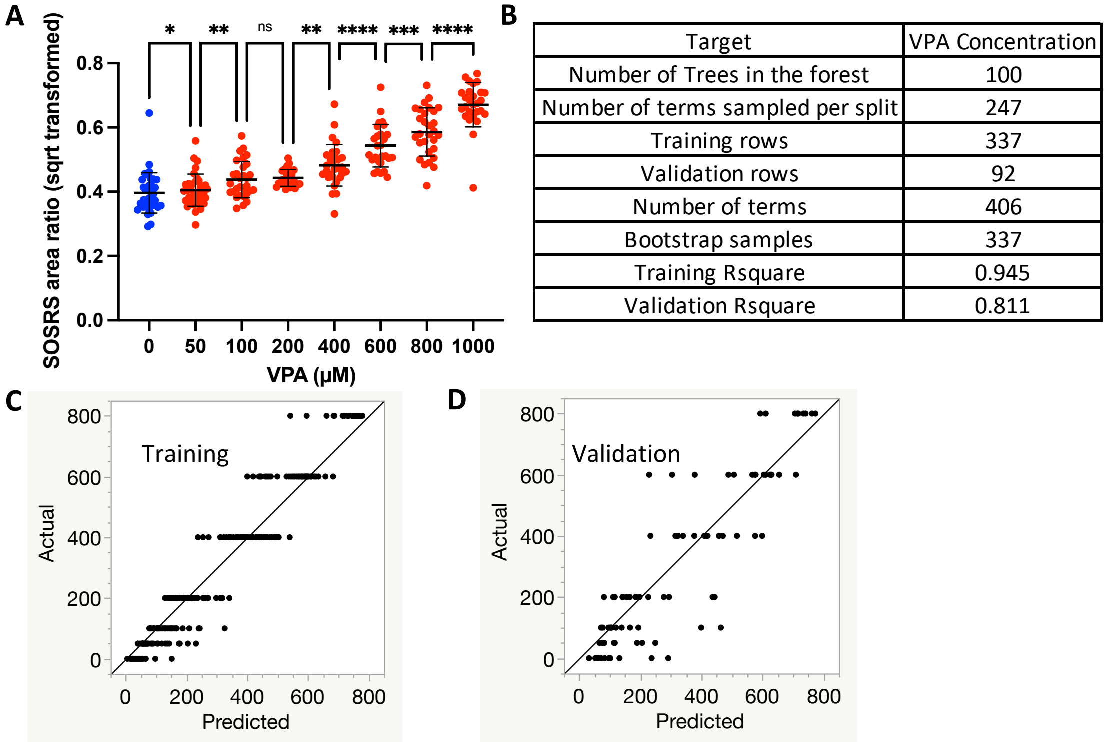

B

Target	VPA Concentration
Number of Trees in the forest	100
Number of terms sampled per split	247
Training rows	337
Validation rows	92
Number of terms	406
Bootstrap samples	337
Training Rsquare	0.945
Validation Rsquare	0.811

E

Term	Number of Splits	SS		Portion
Area_Ratio	65	4203854.01		0.2957
spheroids_RadialDistribution_MeanFrac_GFP_4of6	26	2753430.53		0.1937
spheroids_RadialDistribution_FracAtD_GFP_4of6	25	2688079.63		0.1891
spheroids_RadialDistribution_ZernikeMagnitude_GFP_6_0	6	430509.521		0.0303
spheroids_Intensity_MeanIntensityEdge_CMDR	33	296548.113		0.0209
spheroids_RadialDistribution_FracAtD_GFP_2of6	9	157367.336		0.0111
GFP_Core_AreaShape_Solidity	10	117949.747		0.0083
spheroids_RadialDistribution_ZernikeMagnitude_GFP_7_1	6	112700.192		0.0079
GFP_Core_AreaShape_Compactness	9	93466.0962		0.0066
GFP_Core_AreaShape_Zernike_0_0	9	87516.7566		0.0062
spheroids_RadialDistribution_FracAtD_GFP_1of6	16	83750.4414		0.0059
spheroids_RadialDistribution_MeanFrac_GFP_3of6	7	71758.7242		0.0050
GFP_Core_AreaShape_Extent	13	61462.9614		0.0043
spheroids_RadialDistribution_RadialCV_GFP_2of6	9	59414.21		0.0042
spheroids_RadialDistribution_FracAtD_GFP_3of6	7	58368.3585		0.0041

Supplemental Figure 1. SOSRS lumen area and radial distribution of ZO1-EGFP are distinguishing features of VPA treated SOSRS. (**a**) The normalized lumen area was quantified in SOSRS treated with a dose curve of VPA. Analysis was performed manually. N = 36, 36, 30, 26, 31, 29, 29, and 27, respectively across the 3 independent experiments. (**b**) Specific details and results for the random forest predictive model utilized with the automated data presented in Figure 2B. (**c**) Actual treatment concentration vs. predicted XY scatter-plot based on the bootstrap random forest model for the training data (80% of dataset). (**d**) Actual treatment concentration vs. predicted XY scatter-plot based on the bootstrap random forest model for the validation data (20% of dataset). (**e**) Top 15 most instructive features (of 407 total) for the prediction of VPA treatment concentration (from 50-800 µM). The portion of overall predicted value is the graphs and portion value out of a total of 1.

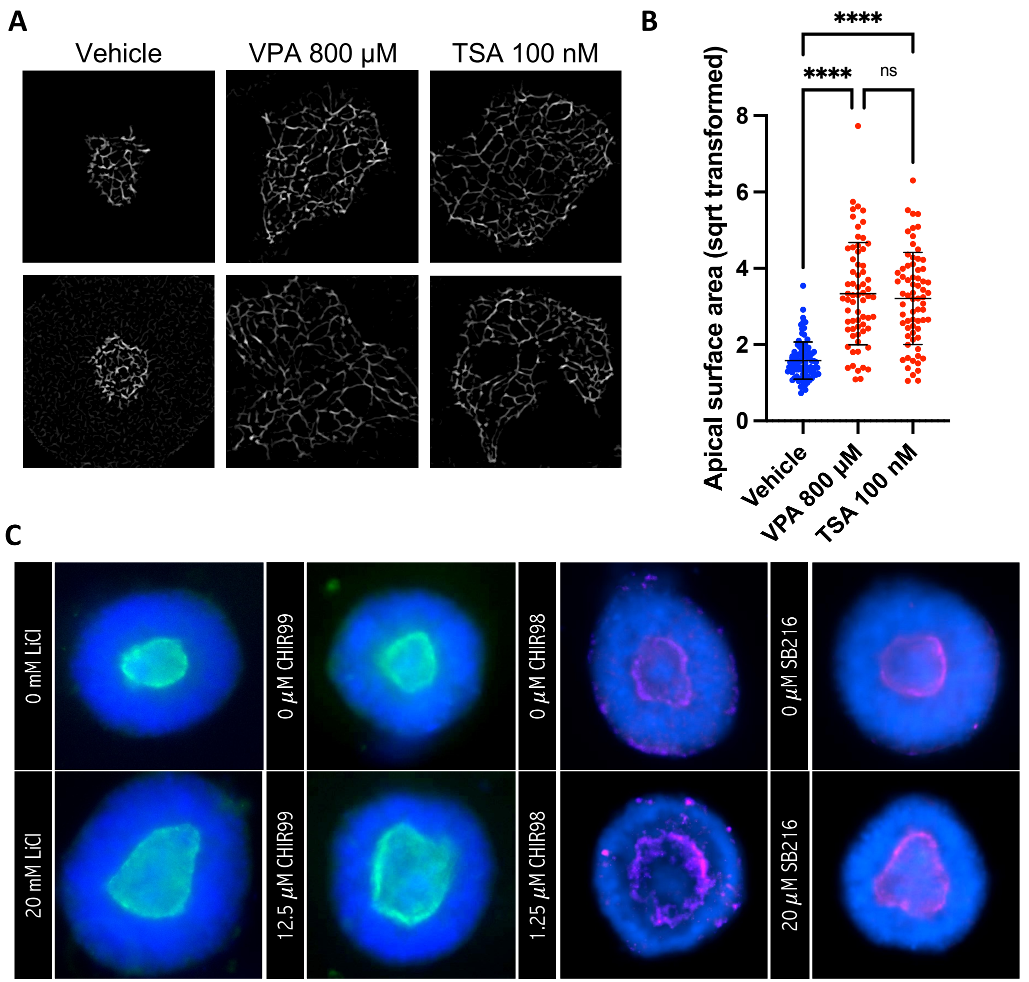

Supplemental Figure 2. HDAC and GSK3β inhibitors decrease apical constriction. (**a**) Apical cell surface areas were imaged by immunostaining for the tight-junction marker ZO-1 which outlines each cells apical surface. Two images for vehicle, 800 μM VPA, and 100 nM TSA are shown. (**b**) Individual surfaces were measured manually for n = 93 across 3 lumens, 66 across 2 lumens, and 69 across 2 lumens, respectively. (**c**) Exemplary manual images of SOSRS taken after labeling for DNA (bis-benzamide) and the apical lumen with either ZO1-EGFP (green) or ZO1 immunostaining (magenta). Immunostaining was necessary for some drugs due to fluorescence in the green channel interfering with ZO1-EGFP signal. Error bars are standard deviation. Statistical analysis performed with Kruskal-Wallis with Dunn's multiple comparisons post hoc. **** $p < 0.0001$.

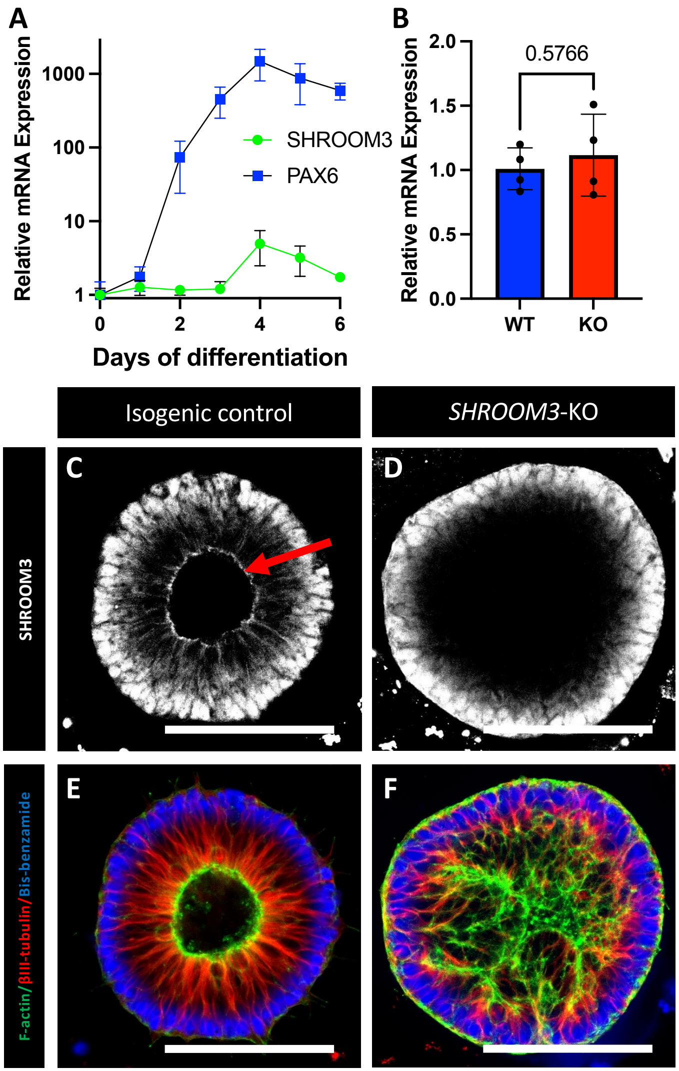

Supplemental Figure 3. Loss of apical SHROOM3 immunostaining confirms loss of functional protein. (**a**) Quantitative reverse transcriptase PCR was performed for *PAX6* and *SHROOM3* across 0-6 days of SOSRS differentiation. N = 2-4 independent differentiations for each data point. (**b**) The same qRT-PCR was performed on day 4 for both the *SHROOM3*-KO line and isogenic control. N = 2 samples for 2 independent experiments (4 total each group). Comparison was by unpaired t-test. Error bars are standard deviation. (**c**-**f**) Confocal micrographs of isogenic (**c**,**e**) and *SHROOM3*-KO SOSRS (**d**,**f**) stained for either SHROOM3 (**c**,**d**) or f-actin, beta-III-tubulin, and bis-benzamide (**e**,**f**). Scale bars are 100 µm.

A

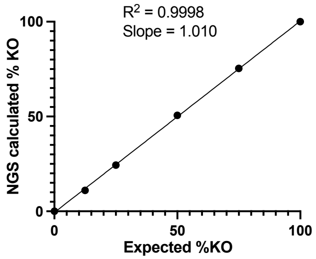

B

Target	F-actin	ZO1
Number of Trees in the forest	100	100
Number of terms sampled per split	150	148
Training Rows	1259	1597
Validation rows	329	416
Number of terms	249	249
Bootstrap samples	1259	1597
Training Rsquare	0.939	0.892
Validation Rsqare	0.832	0.517

C

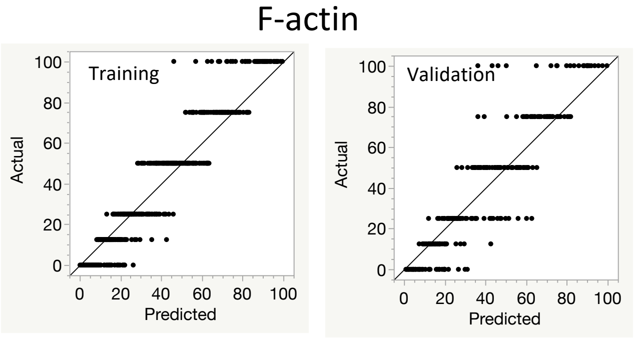

D

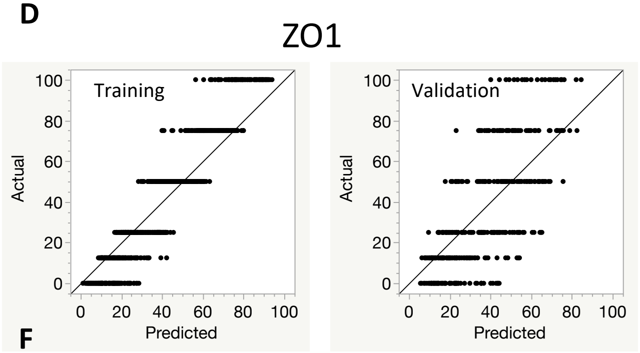

E

Term	Splits	SS		Portion
spheroids_RadialDistribution_MeanFrac_GFP_2of6	45	177894.984		0.2588
spheroids_RadialDistribution_MeanFrac_GFP_3of6	32	64626.1991		0.0940
spheroids_Intensity_MADIntensity_GFP	32	57489.964		0.0836
spheroids_RadialDistribution_MeanFrac_GFP_6of6	28	42843.6168		0.0623
spheroids_Intensity_MeanIntensityEdge_CMDR	27	16003.7817		0.0233
spheroids_RadialDistribution_FracAtD_GFP_2of6	9	15541.1541		0.0226
spheroids_RadialDistribution_FracAtD_CMDR_1of6	20	12028.4781		0.0175
spheroids_Intensity_IntegratedIntensityEdge_CMDR	24	9136.29969		0.0133
spheroids_Intensity_StdIntensityEdge_GFP	16	7931.48711		0.0115
spheroids_RadialDistribution_ZernikeMagnitude_GFP_2_0	14	7005.48256		0.0102
spheroids_Intensity_StdIntensityEdge_CMDR	14	6366.08063		0.0093
spheroids_RadialDistribution_MeanFrac_CMDR_1of6	22	5977.59661		0.0087
spheroids_RadialDistribution_ZernikeMagnitude_GFP_8_0	10	5720.3209		0.0083
spheroids_Intensity_StdIntensity_GFP	10	5624.15682		0.0082
spheroids_AreaShape_HuMoment_6	28	5503.87733		0.0080

F

Term	Splits	SS		Portion
spheroids_RadialDistribution_MeanFrac_GFP_4of6	203	120587.341		0.1392
spheroids_RadialDistribution_FracAtD_GFP_4of6	223	101636.292		0.1173
spheroids_AreaShape_Solidity	298	45975.0403		0.0531
spheroids_RadialDistribution_MeanFrac_GFP_2of6	121	34310.4006		0.0396
spheroids_RadialDistribution_MeanFrac_GFP_1of6	142	19301.0922		0.0223
spheroids_AreaShape_Zernike_6_0	126	12902.3525		0.0149
spheroids_RadialDistribution_RadialCV_GFP_2of6	190	12808.3442		0.0148
spheroids_Intensity_MeanIntensityEdge_CMDR	152	11778.3796		0.0136
spheroids_AreaShape_HuMoment_4	228	11388.8943		0.0131
spheroids_RadialDistribution_RadialCV_GFP_5of6	183	11361.1456		0.0131
spheroids_RadialDistribution_ZernikeMagnitude_GFP_8_4	123	9377.66767		0.0108
spheroids_RadialDistribution_ZernikeMagnitude_GFP_8_0	120	9253.96554		0.0107
spheroids_AreaShape_HuMoment_0	122	8934.72706		0.0103
spheroids_RadialDistribution_FracAtD_GFP_3of6	133	8296.61415		0.0096
spheroids_AreaShape_Compactness	175	7521.03411		0.0087

Supplemental Figure 4. Radial distribution found as most instructive feature to determine SHROOM3 genotype percentage using either f-actin or ZO1 staining. (**a**) Specific details and results for the random forest predictive model utilized with the automated data presented in Figure 6. (**b**) Actual treatment concentration vs. predicted XY scatter-plot based on the bootstrap random forest model for the training data (left: 80% of dataset) and validation data (right: 20% of dataset) for the SHROOM3 SOSRS stained for f-actin (phalloidin-Alexa488). (**c**) Actual treatment concentration vs. predicted XY scatter-plot based on the bootstrap random forest model for the training data (left: 80% of dataset) and validation data (right: 20% of dataset) for the SHROOM3 SOSRS stained for ZO1. (**d**) Top 15 most instructive features (of 407 total) for the prediction of SHROOM3 genotype percentage using f-actin dataset. (**e**) Top 15 most instructive features (of 407 total) for the prediction of SHROOM3 genotype percentage using ZO1 dataset.
